# Beliefs and perceptions of patient safety event reporting in a Canadian Emergency Department: a qualitative study

**DOI:** 10.1007/s43678-022-00400-2

**Published:** 2022-11-07

**Authors:** Trevor Skutezky, Serena S. Small, David Peddie, Ellen Balka, Corinne M. Hohl

**Affiliations:** 1 Department of Emergency Medicine, University of British Columbia, Vancouver, BC Canada; 2 Centre for Clinical Epidemiology and Evaluation, Vancouver Coastal Health Research Institute, Vancouver, BC Canada; 3 School of Communication, Simon Fraser University, Burnaby, BC Canada

**Keywords:** Patient safety, Emergency department, Quality improvement, Sécurité des patients, Service des urgences, Amélioration de la qualité

## Abstract

**Objectives:**

Patient safety events (PSEs) are unwanted or unexpected events that occur during medical care. High cognitive loads and frequent interruptions make emergency departments (EDs) uniquely error prone environments. Yet, frontline clinicians rarely report PSEs using incident reporting systems. The incidence, severity, and preventability of PSEs thus remain poorly understood, and contributing factors are understudied. We sought to understand ED staff beliefs and perceptions about their PSE reporting system and what features they believe are important in such a system.

**Methods:**

We conducted a qualitative study among healthcare providers working in the ED and departmental leadership. We recruited participants via email and held a series of interviews, focus groups, and participatory workshops. We iteratively analyzed the data using the constant comparative method and used thematic analysis to establish themes.

**Results:**

50 participants attended at least one focus group, interview, or workshop. Participants perceived that PSE reporting through formal channels in the ED was challenging. Clinicians had an inherent desire to report PSEs and do so through numerous informal channels, yet underreported in formal reporting systems. The current PSE reporting system did not meet frontline staff needs and was viewed as ineffective in improving care quality and safety. We identified three key features for an improved PSE reporting system: (1) clear definitions; (2) transparency; and (3) simplicity.

**Conclusions:**

In this study, we have identified ideal features for PSE reporting processes to meet the needs of both frontline staff and departmental leadership based on perceptions of current PSE reporting practices. Improved PSE reporting processes have the potential to increase PSE reporting in the ED overall, increasing the availability of information about PSEs to support quality improvement and improve patient safety.

**Supplementary Information:**

The online version contains supplementary material available at 10.1007/s43678-022-00400-2.

## Résumé

**Objectifs:**

Les événements liés à la sécurité des patients (ESP) sont des événements non désirés ou inattendus qui se produisent pendant les soins médicaux. La charge cognitive élevée et les interruptions fréquentes font des services d'urgence des environnements particulièrement propices aux erreurs. Pourtant, les cliniciens de première ligne signalent rarement les ESP à l'aide des systèmes de déclaration des incidents. L'incidence, la gravité et le caractère évitable des ESP restent donc mal compris, et les facteurs contributifs sont peu étudiés. Nous avons cherché à comprendre les croyances et les perceptions du personnel des services d'urgence à l'égard de leur système de déclaration des ESP et les caractéristiques qu'ils jugent importantes dans un tel système.

**Méthodes:**

Nous avons mené une étude qualitative auprès des prestataires de soins de santé travaillant aux urgences et de la direction du service. Nous avons recruté des participants par courrier électronique et organisé une série d'entretiens, de groupes de discussion et d'ateliers participatifs. Nous avons analysé les données de manière itérative en utilisant la méthode comparative constante et avons utilisé l'analyse thématique pour établir des thèmes.

**Résultats:**

50 participants ont assisté à au moins un groupe de discussion, une entrevue ou un atelier. Les participants estiment qu'il est difficile de rendre compte de l'ESP par les voies officielles au sein de l'urgence. Les cliniciens avaient un désir inhérent de signaler les ESP et le faisaient par de nombreuses voies informelles, mais ils étaient sous-déclarés dans les systèmes de déclaration officiels. Le système actuel de déclaration des ESP ne répondait pas aux besoins du personnel de première ligne et était considéré comme inefficace pour améliorer la qualité et la sécurité des soins. Nous avons identifié trois caractéristiques clés pour un système amélioré de rapports sur les ESP : (1) des définitions claires ; (2) la transparence ; et (3) la simplicité.

**Conclusions:**

Dans cette étude, nous avons identifié les caractéristiques idéales des processus de déclaration des ESP afin de répondre aux besoins du personnel de première ligne et de la direction du département, en fonction des perceptions des pratiques actuelles de déclaration des ESP. L'amélioration des processus de déclaration des ESP a le potentiel d'accroître la déclaration des ESP dans les urgences en général, augmentant ainsi la disponibilité des informations sur les ESP pour soutenir l'amélioration de la qualité et améliorer la sécurité des patients.

## Clinician’s capsule

**Table:** 

***What is known about the topic?***
Patient safety events are common during medical care, yet are largely underreported.
***What did this study ask?***
We sought to engage Emergency Department staff and leadership to develop requirements for an improved patient safety event reporting system.
***What did this study find?***
Frontline Emergency Department staff view clear definitions, transparency, and simplicity as key features of patient safety reporting systems.
***Why does this study matter to clinicians?***
Understanding perceptions of patient safety event reporting systems allows for meaningful improvements that may enhance use of the system

## Introduction

Patient safety events (PSEs) are unwanted or unexpected events that occur during medical care, and include near misses, where the incident does not reach the patient; no harm events, where the incident reaches the patient but does not cause harm; and adverse events, where the incident reaches the patient and results in injury, harm, disability, or death [1, 2]. High cognitive loads and frequent interruptions make emergency departments (EDs) uniquely error prone environments, threatening patient safety and reducing care quality [3–7]. Furthermore, EDs are overcrowded and understaffed, increasing risks to patients and clinical burden on staff [8–10].

In 2007, British Columbia’s (BC) health authorities began implementing Patient Safety and Learning System (PSLS), a privately developed, web-based incident reporting software, to capture PSEs and identify system factors to prevent their recurrence [11]. Clinicians report in PSLS voluntarily using electronic forms that capture information about different incidents (e.g., falls), including a description of the event and its impact, contributing factors, patient and reporter information, and follow-up actions taken. Staff must submit incident reports, as defined in the Disclosure of Patient Safety Incidents policy, to PSLS per the Incident Management policy [12, 13]. Training and reporting guidelines are available on the staff Intranet. After clinicians submit reports, supervisors are responsible for reviewing and coordinating the investigation of incidents, including following up with the reporter either one-on-one, in meetings, or through group communications to close the loop on reporting [13].

Studies indicate underreporting is commonplace in voluntary incident reporting systems despite institutional policies [14–17]. Fear of punitive action, poor safety culture, and time required to complete forms are primary deterrents to reporting [2, 15, 18–21]. Unique challenges in the ED may compound these issues [3–7]. While previous investigators have studied barriers to PSE reporting and suggested that end-user engagement may improve reporting platforms, few studies have sought to understand limitations of and improve upon existing PSE reporting processes from the perspective of ED staff and departmental leadership at an acute care setting in Canada [11, 22–25]. The primary objective of this study was to explore the beliefs and perceptions of ED staff and leadership of the current PSE reporting system and identify features that they believe are important in such a system.

## Methods

### Study design, setting, and time period

We conducted a qualitative study using semi-structured focus groups, interviews, and workshops at Vancouver General Hospital (VGH), a large academic urban hospital with an annual ED census of 95,000 patients, between August 2017 and May 2019 in a research office on site [26]. 68 physicians, 60 residents, and 260 nurses work in the ED.

We approached this study through the philosophical and analytical framework of constructivist grounded theory, recognizing the researcher’s role in the co-construction of knowledge [27]. We used the Consolidated Criteria for Reporting Qualitative Research (COREQ) checklist to ensure a robust study [28]. We received ethics approval from the University of British Columbia Behavioural Research Ethics Board (H16-01238).

### Recruitment

VGH ED nurses, physicians, and departmental leaders were eligible to participate. Using convenience sampling, we approached eligible individuals at VGH to participate in a focus group or interview. We sought to sample a representative distribution of clinical staff based on staffing in the ED and aimed to continue recruitment until we reached data saturation, but pragmatically recruitment ceased when no additional individuals volunteered within a reasonable timeframe. A pre-existing professional relationship with participants who worked in the ED facilitated recruitment. We sent study information to those who agreed to participate via email, which informed prospective participants of the study purpose. At the completion of each focus group or interview, we invited participants to attend a workshop.

### Data collection

We prepared two semi-structured focus group and interview protocols, one for frontline staff and one for departmental leadership, informed by a review of relevant literature completed prior to study initiation. We used our protocol to guide the sessions, capturing emerging topics and ideas to explore in subsequent sessions (Appendix 2).

The principal investigator (TS), an emergency medicine resident, received methodological guidance and training from a social scientist on the study team (EB) with qualitative expertise. The principal investigator (TS) led the focus groups and interviews. A research assistant (DP) attended some sessions to co-facilitate and take notes that highlighted emerging topics and areas for further exploration in subsequent sessions. At the beginning of each session, we reiterated study goals and introduced the researchers. We continued each focus group or interview until we reached data saturation, ranging from 45 min to 1.5 h. Toward the conclusion of the focus group and interview portion of the study, we felt we achieved saturation as no new concepts or ideas began to emerge.

We developed a list of ideal features for a PSE reporting based on focus group and interview findings, which we presented to workshop participants. We used feedback to iteratively refine and finalize the list of ideal features. We audio recorded all interviews and focus groups, which a professional transcriptionist transcribed for analysis.

### Data analysis

We coded and analyzed transcriptions using NVivo 12 (QSR International, version 12, 2020). We employed an inductive, grounded method to develop the coding structure and utilized the constant comparative method to incorporate relevant data within the final coding structure [29]. We referred to field notes to identify emerging themes from the sessions. Three researchers (TS, DP, SSS) met intermittently to refine the coding structure until the final structure was agreed upon (Appendix 3). We then recoded all transcripts using the final coding structure, which organized participant comments along the following themes: factors affecting PSE reporting behaviour; medium of PSE reporting; and, components of an ideal system. Two researchers (TS, DP) coded and compared 20% of the transcripts to ensure that the application of coding structure was consistent. We used thematic analysis to establish themes [30, 31].

## Results

### Focus group, interview, and workshop participant characteristics

Fifty participants attended at least one focus group, interview, or workshop. Each focus group had 2–5 participants, while interviews were one-on-one. We held two large group workshops with 28 and 9 participants, respectively. Nine individuals participated in both a focus group or interview and a workshop. We gathered information on participant’s training and years in practice for analytical purposes (Table 1).

**Table 1:** Characteristics of focus group and workshop participants

Characteristics of Participants	Initial Focus Group and Interview Participants (*N* = 22)	Workshop participants (*N* = 37)
Training	*n* (%)	*n* (%)
Emergency resident	8 (36)	15 (41)
Emergency nurse	7 (32)	13 (35)
Emergency physician	7 (32)	9 (24)
Years in practice		
Emergency Residency	8 (36)	15 (41)
≤ 5 years	3 (14)	4 (11)
6–10 years	4 (18)	1 (3)
11–20 years	3 (14)	2 (5)
21–30 years	0 (0)	3 (8)
> 30 years	1 (5)	4 (11)
No response	3 (14)	8 (22)
Other professional activities		
Clinical sole focus	1 (5)	9 (24)
Residency	8 (36)	15 (41)
Research	1 (5)	3 (8)
Administration	7 (32)	8 (22)
Education	2 (9)	1 (3)
Medicolegal	1 (5)	0 (0)
No response	2 (9)	1 (3)

### Beliefs and perceptions of PSEs and current reporting practices

In focus groups and interviews, we sought to understand beliefs and perceptions about current PSE reporting practices. We describe our three main findings with supporting quotations.

#### PSEs are common but poorly defined

Staff were unaware of guidelines that define PSEs and differed in what they believed constituted a reportable event (Table 2). Participants generally agreed that events that led to direct harm and near misses should be reported, however, some believed that their colleagues might not perceive near misses as worth reporting.

**Table 2:** Representative participant quotes regarding definition of PSEs

*“*anything that endangers or potentially endangers a patient and/or anything that endangers or potentially endangers a staff member.” (Physician) “It shouldn’t have to be a major case for us to actually collect data about it.” (Physician) “so the concept of near miss doesn’t seem to be something that people – as a way of improving and preventing an actual miss—they don’t see it that way” (Physician) “So an emerging problem to me or like a blatant error needs to be addressed. If it's something that we already know about and we battle regularly like wait time for consultants and there is tons going into that, I'm not sure what an SLS helps for that.” (Physician) “You know I know nothing about like what events do they want to hear about? What events do they not want to hear about?” (Resident) “I remember somewhere there was an objective set of criteria but a lot of the time it's subjective and it's either near misses or misses.” (Nurse)	

Participants identified several examples of reportable events (Appendix 1). There was consensus that medication errors, falls, and violence constitute reportable PSEs, representing three of the four most commonly discussed PSEs. These are easily identifiable events, and have been the focus of patient safety initiatives, particularly among frontline nurses. As one departmental leader noted: “we really were encouraging reporting of falls.” Participants also frequently cited operational challenges as PSEs, but were unsure if a delay in care should trigger a report.

There was less consensus about whether to report system-level issues, like overcrowding. Physicians were more likely to view these as PSEs, but rarely reported them in PSLS. Participants acknowledged that conditions that reduce care quality may constitute PSEs, but their frequency made it difficult for staff to differentiate between suboptimal conditions of a busy ED and a PSE. One physician commented: “it happens so frequently it's hard to call it [a PSE] because it becomes normal but it's not okay.” Among all groups, identifying reportable events did not necessarily translate into a completed report.

#### Patient Safety Learning System is underutilized and perceived as not meeting clinician needs

Most participants were aware of PSLS as a PSE reporting tool, and some had used PSLS, although no resident physicians had. Many of those who had used it had negative experiences citing rigid, time-consuming forms with unfamiliar fields that do not fit clinical workflows (Table 3).

**Table 3:** Participant quotes about beliefs and perceptions of PSLS

“so I have some experience with using the PSLS. The frequency of using it is not very high compared to the number of times I've encountered safety concerns.” (Nurse) “never would we have seen that in trending from SLS” (Departmental Leadership) “nobody knows anything about [PSLS] other than you're meant to do a PSLS if something bad happens” (Nurse) “…my understanding is that PSLS is for risk management not for quality improvement.” (Physician) “that system is known to be quite lengthy and nothing gets reported” (Departmental Leadership) “the SLS forms are an issue” (Nurse) “So meaningful outcome from the PSLS in my experience has been very little and when I have checked in with colleagues that seems to be the feeling, is that what we would like is to have it addressed like seriously and look at what can we change, and instead it feels like a bit of a brush-off.” (Physician) “one the biggest problems with any of these systems, and I've worked in many different institutions, is that you fill out a form but you have no feedback” (Nurse) “whenever it comes to the SLS and I have to write my name, I always feel it's a punitive process.” (Nurse) “but I think that the SLS is just such a cumbersome program that [frontline staff] can't figure out how to fit that into their other duties” (Departmental Leadership) “There are too many fields I think.” (Nurse) “however I don’t like the double charting” (Nurse) “So people think well, why do I bother – why would I bother to report this? This has been reported on 20 different times.” (Physician) “They're not going to change anything.” (Physician)	

Nurses expressed frustration with the redundancy of PSLS that necessitated duplicate charting. Physicians viewed PSLS as more of a risk management tool than a mechanism for quality improvement. Staff did not see how PSE reporting in PSLS translated into an improved work environment or patient care, creating a sense of futility. Although departmental leadership used PSLS to follow up on reported events, they acknowledged its lack of functionality for frontline staff.

#### Participants want to and do report PSEs through informal channels

Staff viewed informal PSE reporting as a professional responsibility and a tool to facilitate change by directing attention to emerging problems, informing education, and engaging staff in improvement efforts. These factors motivated staff to report PSEs through informal channels that evolved organically to meet these needs, including emails, verbally, and in nursing notes (Table 4). The perceived duty to report did not extend to PSLS, which staff bypassed for all but the most serious events.

**Table 4:** Real-world PSE reporting practices

“It is very haphazard. It is multiple forms, whether it be email, text, in person, not usually notes but on PSLS, in a group meeting, a team leadership meeting what-have-you, in part because there's so many different ways the information flows in our department, right.” (Departmental Leadership) “it's too much of a mix of how the information gets fed through and up.” (Departmental Leadership) “I just – I don’t really report very much anymore. I used to a lot as a new grad and I found it like very cumbersome to go through that whole process. So I've just – I hardly do them anymore. Yeah, if I have an issues, then I'll like, talk to leadership directly.” (Nurse) “I think [frontline staff] definitely are motivated to see changes and – but I think that the SLS is just such a cumbersome program that they can't figure out how to fit that into their other duties.” (Departmental Leadership) “I'm going to do this because I'm a responsible nurse” (Nurse) “…an email—will [sic] take that and then review the case and then decide if it's pursuable, not pursuable or if there's a learning element that can be brought up to the rest of the group. And then they will use that case maybe for our monthly seminars-slash-rounds on patient safety and quality improvement rounds.” (Departmental Leadership) “Nursing notes get photocopied and it gets put under the door, my door because that happened at night shift.” (Departmental Leadership) “I documented it on the chart, not in any system” (Physician) “I have successfully delegated a nurse to complete it [in PSLS] because I felt that they had more time available than I did” (Physician) “In the couple of incidences that I thought were critical I did speak to the charge nurse about the fact that I think this needs to go somewhere” (Physician) “Yeah so there’s a bit of confusion I think about what the actual purpose is.” (Resident)	

### Perceived features of an ideal PSE reporting system

Informed by participants’ beliefs and perceptions about PSEs and current reporting practices, we subsequently captured perspectives about the features of an ideal PSE reporting system. We describe the three main features below, and summarize supporting quotations in Table 5.

**Table 5:** Features of an ideal PSE reporting system

Feature	Representative Quotes
Clear definition	*“If you know that there is a process you are more likely to follow the process.”* (Physician Leadership) *“people need a robust education and understanding of why they are doing something.”* (Nurse)
Transparency about what reporting does	*“If people were to see the results of SLS reports in activity and actually having an end result, I think that would get a lot more buy-in.”* (Departmental Leadership) *“if I take the time to do a PSLS I want to know where it went, what their processes of decision making were in terms of deciding it's important or it's not important. I want to see a decision process and I'd like to see the end results of what came about from it”* (Physician) *“If I was confident that there would be some direct feedback or result or patient advocacy I would invest as much time as needed.”* (Physician)
Simplify reporting form	*“[it would be] my dream system if I could do a [P]SLS as fast as I could order an ECG. Six clicks and one that minimizes duplication”* (Nurse) *“…it would be easier to use if it was more streamlined in its design, fewer fields, less laborious information where you could just input the fields that you felt were relevant”* (Physician) *“I’d just want to know the bare numbers in the end. I don’t need to know every detail about it.”* (Departmental Leadership)

#### Provide clear definition of PSEs

Participants acknowledged a need for shared understanding and clear definition of what constituted reportable PSEs among staff and departmental leadership to support reporting. Participants suggested that including event type classification on PSE reporting forms may increase clarity about what constitutes a reportable event, and that broader communication from departmental leaders may increase PSE identification and reporting among staff.

#### Provide transparency about what reporting does

Staff want to share information that will lead to improved patient safety and care quality, and departmental leadership want to receive this information, but many believed that current PSE reporting channels lack sufficient transparency to demonstrate improved outcomes following reporting. Participants want transparency about what reporting does and were interested in being able to indicate their preferred level of feedback within the reporting form to clarify expectations and increase reporting.

#### Simplify the reporting form

Participants felt that PSE reporting should be simple and easy to use with free-text options and few mandatory fields, and that leveraging existing informal reporting channels may reduce duplication. Many acknowledged that detailed reporting requirements are a time burden and barrier to reporting, yet some managers noted the need for sufficient data to determine next steps, follow up, and generate feedback. Participants agreed that an ideal reporting form should balance simple data entry while enabling further escalation and investigation if needed.

## Discussion

### Interpretation of findings

In this study, we explored ED staff and leadership beliefs and perceptions of PSE reporting. As in other similar settings, PSEs in our ED are likely underreported. Beliefs and perceptions about the PSE reporting system, including low awareness of the system itself, are contributing factors to its underuse. Despite a willingness to report PSEs and receive PSE reports, the current system fails to meet clinicians’ needs and lacks clarity around reporting processes and actions taken. Participants identified features of an ideal PSE reporting system, which may increase usage of and engagement with the current system in capturing PSEs, if incorporated. Creating clear definitions of PSEs and reporting guidelines may support clinician understanding of reportable event types. Establishing closed feedback loops may clarify how and why clinicians’ reports affect decision-making. Streamlining and simplifying the reporting process may reduce the burden of documentation among busy frontline providers. Ultimately, the ideal PSE reporting form must balance leadership’s information needs for quality improvement with frontline ED staff’s limited time.

### Comparison to previous studies

Several past studies described barriers to PSE reporting that are consistent with our study. A 2011 study identified six barriers to PSE reporting, which included inaccessibility of reporting forms [18]. A related study on adverse drug events observed that providers documented events in charts to support continuity of care but never reported them to external agencies through formal reporting channels, citing time constraints and duplication [19].

Previous studies have also identified features that facilitate PSE reporting that are consistent with our study, including clear reporting guidelines, usable reporting systems, and feedback loops to create learning opportunities [2, 20]. Previous investigators suggested engaging frontline healthcare providers in design processes to improve reporting and enable systematic data collection required to advance patient safety in EDs [32]. In keeping with prior suggestions, our study adds to the literature a nuanced understanding of frontline clinicians’ and departmental leaderships’ perceived ideal features of a PSE reporting system, which aims to address reporting barriers and underreporting in the ED context.

### Strengths and limitations

A strength of this study is recruitment of participants with diverse clinical backgrounds, time in practice, and roles in the ED, including ED leadership, resulting in a diversity of perspectives. We sampled to thematic saturation by completing focus groups and interviews until no new themes emerged, and we returned concepts to staff multiple times to refine themes and ensure we interpreted data correctly.

The context of the study may have affected participant responses. Some participants knew the interviewer. Furthermore, the group setting may have been intimidating to some participants, causing them to be less forthcoming with their answers.

Individuals who agreed to participate are more likely to be interested in PSE reporting, and thus may have been more familiar with the PSE reporting process, leading to bias. We sampled a small proportion of staff from a single ED, which may limit the generalizability and transferability of our findings to other settings; however, this was not a primary goal of our study. Residents, who were slightly over-represented, were less familiar with PSE reporting processes, which may have affected findings.

Being an emergency physician, the principal investigator undertook the project with an understanding of responsibilities toward patient safety and expected use of PSLS. We mitigated the risk of bias and increased the credibility of our study by including team members with different disciplinary backgrounds in the research project. In particular, having three researchers develop the final coding structure and two researchers coding 20% of the transcripts reduced the risk of subjectivity bias and increased reliability.

### Clinical implications

Current PSE reporting systems do not meet the needs of the frontline staff who are responsible for reporting, and consequently, fail to meet the data needs of departmental leadership. This study has allowed us to understand the needs of ED staff regarding PSE reporting. If this data is used to streamline PSE reporting processes it may lead to increased patient safety and quality of care.

### Research implications

Future work should seek an improved understanding of which features of PSE reporting systems are associated with increased use by ED staff, and which features support the identification of local priorities and action items.

## Conclusion

This study reveals that ED staff view clear definitions, transparency, and simplicity as key features of a PSE reporting system. While operationalizing these findings may support PSE reporting in the ED, other systemic barriers must be addressed to generate comprehensive patient safety data in the ED that can be used for surveillance, performance assessment, and to identify new or uncommon sources of harm. Only then will the ED foster an environment of patient safety, clinical excellence, and staff satisfaction.

## Supplementary Information

Below is the link to the electronic supplementary material.

Supplementary file1 (PDF 654 kb)

Supplementary file2 (PDF 45 kb)

Supplementary file3 (PDF 81 kb)

## References

[1] Institute of Medicine (US) Committee on Quality of Health Care in America. To Err is Human: Building a Safer Health System. Washington (DC): National Academies Press. 2000. 25077248

[2] Health Quality Ontario (2017). Patient safety learning systems: a systematic review and qualitative synthesis. Ont Health Technol Assess Ser.

[3] Croskerry P, Sinclair D (2001). Emergency medicine—a practice prone to error?. CJEM.

[4] Fordyce J, Blank FS, Pekow P, Smithline HA, Ritter G, Gehlbach S (2003). Errors in a busy emergency department. Ann Emerg Med.

[5] Brennan TA, Leape LL, Laird NM, Hebert L, Localio AR, Lawthers AG, et al. Incidence of adverse events and negligence in hospitalized patients. Results of the Harvard Medical Practice Study I. N Engl J Med. 1991;324(6):370–6. 10.1056/NEJM199102073240604 10.1056/NEJM199102073240604 1987460

[6] Leape LL, Brennan TA, Laird N, Lawthers AG, Localio AR, Barnes BA, et al. The nature of adverse events in hospitalized patients. Results of the Harvard Medical Practice Study II. N Engl J Med. 1991;324(6):377–84. 10.1056/NEJM199102073240605 10.1056/NEJM199102073240605 1824793

[7] Baker GR, Norton PG, Flintoft V, Blais R, Brown A, Cox J (2004). The Canadian adverse events study: the incidence of adverse events among hospital patients in Canada. CMAJ.

[8] Azpiri J. (2022). Staff shortages lead to temporary emergency department closures at 3 rural B.C. hospitals. Available from: https://www.cbc.ca/news/canada/british-columbia/bc-rural-emergency-room-closures-1.6470405. Accessed July 22, 2022.

[9] Wright T (2022). ‘We are absolutely destroyed’: health workers facing burnout, even as COVID levels ease. Available from: https://globalnews.ca/news/8889103/covid-burnout-destroyed-health-workers/. Accessed July 22, 2022.

[10] Sun BC, Hsia RY, Weiss RE, Zingmond D, Liang LJ, Han W, McCreath H, Asch SM (2013). Effect of emergency department crowding on outcomes of admitted patients. Ann Emerg Med.

[11] British Columbia Patient Safety and Learning System. BC Patient Safety and Learning System (BCPSLS) Evaluation Report. January 25, 2008. Available from: https://bcpslscentral.ca/wp-content/uploads/2014/04/PSLSEvaluationReport_FINAL_Jan2508_website1.pdf. Accessed 1 Oct 2021.

[12] Vancouver Coastal Health. Disclosure of patient safety incidents (D-00-11-30094). Vancouver Coastal Health. August 10, 2021. Available from: http://shop.healthcarebc.ca/vch/VCHPolicies/D-00-11-30094.pdf. Accessed 22 July 2022.

[13] Vancouver Coastal Health. Incident management (Patient/Client/Resident) (D-00-11-30018|CA_700). Vancouver Coastal Health. August 17, 2016. Available from: http://shop.healthcarebc.ca/vch/VCHPolicies/D-00-11-30018.pdf. Accessed 22 July 2022.

[14] Department of Health and Human Services, Office of Inspector General. Hospital incident reporting systems do not capture most patient harm. Washington, DC; 2012. http://oig.hhs.gov/oei/reports/oei-06-09-00091.pdf. Accessed 24 Mar 2022.

[15] Uribe CL, Schweikhart SB, Pathak DS, Dow M, Marsh GB (2002). Perceived barriers to medical-error reporting: an exploratory investigation. J Healthc Manag.

[16] Lawton R, Parker D (2002). Barriers to incident reporting in a healthcare system. Qual Saf Health Care.

[17] Milch CE, Salem DN, Pauker SG, Lundquist TG, Kumar S, Chen J. Voluntary electronic reporting of medical errors and adverse events. An analysis of 92,547 reports from 26 acute care hospitals. *J Gen Intern Med.* 2006;21(2):165–70. 10.1111/j.1525-1497.2006.00322.x 10.1111/j.1525-1497.2006.00322.x PMC1484668 16390502

[18] Brubacher JR, Hunte GS, Hamilton L, Taylor A (2011). Barriers and incentives for safety event reporting in emergency departments. Healthc Q.

[19] Hohl CM, Small SS, Peddie D, Badke K, Bailey C, Balka E (2018). Why clinicians don't report adverse drug events: qualitative study. JMIR Public Health Surveill.

[20] Burlison J, Quillivan R, Kath L, et al. A multilevel analysis of U.S. hospital patient safety culture relationships with perceptions of voluntary event reporting. J Patient Saf. 2020;16 (3): 187–193. 10.1097/PTS.0000000000000336. 10.1097/PTS.0000000000000336 PMC5415419 27820722

[21] Tevis S, Schmocker R, Wetterneck T (2020). Adverse event reporting: harnessing residents to improve patient safety. J Patient Saf.

[22] Peddie D, Small S, Wickham M, Bailey C, Hohl C, Balka E (2017). Designing novel health ICTs to support work, not generate it: five principles. Stud Health Technol Inform.

[23] Peddie D, Small SS, Badke K, Bailey C, Balka E, Hohl CM (2018). Adverse drug event reporting from clinical care: mixed-methods analysis for a minimum required dataset. JMIR Med Inform.

[24] Chruscicki A, Badke K, Peddie D, Small S, Balka E, Hohl CM (2016). Pilot-testing an adverse drug event reporting form prior to its implementation in an electronic health record. Springerplus.

[25] Hohl C, Lexchin JR, Balka E (2015). Can reporting of adverse drug reactions create safer systems while improving health data?. CMAJ.

[26] VGH+UBC Hospital Foundation. 2014. Available from: https://vghfoundation.ca/2014/03/03/did-you-know-quick-facts-about-vghs-emergency-department/. Accessed March 24, 2022.

[27] Charmaz K (2006). Constructing grounded theory: a practical guide through qualitative analysis.

[28] Tong A, Sainsbury P, Craig J (2007). Consolidated criteria for reporting qualitative research (COREQ): a 32- item checklist for interviews and focus groups. Int J Qual Health C.

[29] Noble H, Mitchell G (2016). What is grounded theory?. Evid Based Nurs.

[30] Guest G, MacQueen KM, Namey EE. Introduction to applied thematic analysis. In: Applied Thematic Analysis. Thousand Oaks, CA: SAGE Publications; 2012:3–20.

[31] Chapman AL, Hadfield M, Chapman CJ (2015). Qualitative research in healthcare: an introduction to grounded theory using thematic analysis. J R Coll Physicians Edinb.

[32] Okafor NG, Doshi PB, Miller SK, McCarthy JJ, Hoot NR, Darger BF (2015). Voluntary medical incident reporting tool to improve physician reporting of medical errors in an emergency department. West J Emerg Med.

