# Supplementary material for: Beliefs and perceptions of patient safety event reporting in a Canadian Emergency Department: a qualitative study
Source: CJEM. 2022 Nov 7;24(8):867–75. doi: 10.1007/s43678-022-00400-2 (PMC9763130; doi:10.1007/s43678-022-00400-2)
Supplement: Supplementary file 1 — Supplementary file1 (PDF 654 kb) [file 43678_2022_400_MOESM1_ESM.pdf]

<u>Emergency Department Patient Safety Event (PSE) Reporting</u>

Discussion Guide for <u>Leadership Team</u> Focus Groups

I. Welcome and Introductions

II. Guidelines

- No right or wrong answers, just different points of view
- Session will be recorded; reminder of confidentiality; verbal consent script
    - Confirm participants are comfortable with recording
- Listen respectfully; only one person speaks at a time
- Ensure cell phones are on vibrate
- We will be asking you questions about Patient Safety Events (PSEs)

III. Introduce Topic

- Broadly, interest is in improving information sharing about PSEs. Open to suggestions about how that is best achieved.
- One approach is reporting (via systems like PSLS) and we will solicit thoughts on reporting and how it can be improved, but we don't want to limit conversation to this approach.
- Mostly interested in your perspectives as leadership, but feel free to speak from clinical experience as well.

IV. Questions

A. Baseline perceptions of patient safety events
    a. What do you perceive to be the most common, serious, or preventable care-related risks to patients in the ED?
    b. What factors contribute to PSEs? Consider factors across individual, organizational, and system levels.
    c. How is information about PSEs currently shared between clinical staff and leadership? Do you think this communication is adequate? Does it result in tangible improvements to patient safety?
    d. Are frontline clinicians interested in communicating PSEs to leadership?

B. Experiences with patient safety events
    a. How has the ED acted to address PSEs in the past?
    b. What types of information sharing led to this action? (*e.g., verbal complaints, reports, media attention?*)
    c. How do you usually become aware of PSEs or potential emerging risks to patient safety in your department?

C. Baseline perceptions of reporting

a. What are your impressions of PSE reporting as a method of information sharing? Do you think it is effective at improving patient safety?
b. What limits PSLS use by frontline clinicians?

D. Experiences using PSLS in administrative role
   a. How do you use the PSLS in your role?
   b. Do you find PSLS a useful tool in your daily practice?
   c. What information that is currently reported through the PSLS is not used? In contrast, what information is the most useful?
   d. How do you use the information reported through PSLS?
   e. What are your thoughts on the user-friendliness and usefulness of the current interface (front-end and back-end)? What are the best features of the existing system?
      If No
      i. What other processes do you use to learn about PSEs in your department?

Imagine for a moment that you are tasked with designing a PSE communication process to be used in the ED.

E. Questions about ideal state of PSE communication
   a. What events that occur over the course of patient care do you want to front-line staff to communicate? Do you have any interest in broadening the scope of PSEs that staff are encouraged to communicate to leadership? *(e.g. delay to patient care, computer problems, delay from consulting services)*
   b. What specific information do you want shared about a given PSE? Why?
   c. What do you think is the best way for front-line staff to share information related to PSEs? Do you have any new ideas for how better information sharing could be achieved? *(current PSLS system, modification of the current system, verbal, on own device etc.)*
   d. How much front-line staff time should be used to share information about PSEs? (*e.g. how much time spent reporting, discussing, developing solutions*)
   e. Would you be willing to do more work on your end if it meant that front-line staff spent less time reporting PSEs? *(PSE communication takes more of your time but less of their time?)*
   f. Is there anything that you think could be done to increase the likelihood that staff
      i. share information about PSEs with leadership?
      ii. report PSEs using the PSLS system?

F. Questions about ideal state of PSE data
   a. Is there anything that you think could be done to make PSE data more useful in your job?
   b. How to you want to receive information from front-line staff regarding PSEs?
      i. What form do you want that data to be presented in? *(cumulative data vs individual events)*

c. Is there anything that you think could be done to streamline the handler process?
d. Is there anything that you think could be done to close the loop with front-line staff when they report a PSE? What information do you think staff would benefit from receiving back from the PSE reporting system?
e. Is there any role for PSE data in the accreditation process? *(Could we collect this data in real-time to make accreditation easier?)*

G. Wrap up and Debrief
   a. Summarize and review responses
   b. Discuss purpose of project and next steps (what will be done with this information, will participation be requested in the future)
   c. Reminder to keep our discussion confidential
   d. Provide contact information for study team
   e. Thank participants

<u>Emergency Department Patient Safety Event (PSE) Reporting</u>

Discussion Guide for <u>Front-Line Staff</u> Focus Groups

Welcome and Introductions

Guidelines

- No right or wrong answers, just different points of view
- Session will be recorded; reminder of confidentiality
- Listen respectfully; only one person speaks at a time
- Ensure cell phones are on vibrate
- We will be asking you questions about Patient Safety Events (PSEs)

Verbal consent script

Introduce Topic – we're talking about PSE reporting in the ED.
Questions – **Front-Line Staff Group**

A. Questions about experience with PSEs

    a. What do you consider to be a PSE?
    (Prompt: All events that impact patient care, near miss events, events that delay patient care, other ideas?)

B. Questions about PSLS use behaviour. *Thinking about a PSE that you experienced in the past:*

    a. If you have reported a PSE using the PSLS, what was your motivation for reporting?

    b. If you have reported a PSE using the PSLS, what physical factors contributed to your decision to report it in PSLS? What factors made the process more difficult *(computer access, knowledge of the PSLS system)*

    c. What are the real and perceived barriers to reporting PSEs?

    (Prompt: Fear of punishment/discipline, difficult reporting form, time consuming, feeling of futility; i.e. reporting won't make a difference, no feedback from the system, unsure of reporting requirements/what constitutes a PSE)

C. Questions about the PSLS user experience:
    a. If you did report the PSE using the PSLS, what was your experience?

D. Questions about ideal reporting state:

a. Is there anything that could be done to increase the likelihood that you would report a PSE?

b. Would you be willing to report more frequently if it were easier and less time consuming?

Imagine for a moment that you could decide how PSEs are managed in the Emergency Department (ED). You are tasked with designing how front-line staff such as yourself report PSEs. With that in mind:

a. What type of events do you think should be reported?
(None, all events that impact patient care, near miss events, events that delay patient care, other ideas?)

b. How would you prefer to report PSEs?
(Not at all, verbally, on a free-standing iPad, on your own device, other options)

c. What is the maximum amount of time you would spend to report a PSE?

d. Where in your clinical workflow would you want to interface with a PSE reporting system?

e. If you could track one thing that you believe would have an impact on patient care, what would it be?

f. What feedback have you experienced after submitting a PSE?

g. Do you want to receive feedback from the reporting system when you report a PSE?
    i. *[If yes]*
        1. What kind of feedback do you want?
        (Acknowledgment of submission, a thank you, outcome of report/findings, planned changes or education seminars stemming from the submission)
        2. What kind of follow up do you want?
        3. What kind of feedback/follow up would help you improve your practice?
    ii. *[if no]* – why not?

Conclusion: Wrap up and Debrief. Re-affirmation of confidentiality. Provision of contact information for study team.
