# Supplementary material for: Beliefs and perceptions of patient safety event reporting in a Canadian Emergency Department: a qualitative study
Source: CJEM. 2022 Nov 7;24(8):867–75. doi: 10.1007/s43678-022-00400-2 (PMC9763130; doi:10.1007/s43678-022-00400-2)
Supplement: Supplementary file 2 — Supplementary file2 (PDF 45 kb) [file 43678_2022_400_MOESM2_ESM.pdf]

Name of Code	Node	Sub-node	Sub-node	Sub-node	Description
**Current state of PSE reporting**					General statements, not captured or not relevant to a single subcategory
	**Factors affecting reporting behaviour**				Factors influencing reporting across multiple areas/not captured in other categories
		ED culture and leadership			Social behavior/norms/habits in ED influencing reporting behaviour.
		Clarity of Process			Staff awareness of why, how, when, what, where influencing reporting behaviour.
		User-friendliness of Process			Relative ease of process, through any medium influencing reporting behaviour.
		Time Burden and Priorities			Time available/priority influences reporting behaviour.
		Value of reporting			Value/utility&absence of value/utility influences reporting. General statements.
			Front Line		Value/utility&absence of value/utility influences reporting behaviour
				Feedback	Feedback/absence of feedback to reporter influences reporting behaviour.
				Action	Actions perceived to result from reports by staff influences reporting behaviour.
				Data	Generation/potential generation of data influences reporting behaviour.
			Leadership		Value/utility&absence of value/utility influences reporting of by leadership
		Potential consequences			...of PSE and to staff as a result of reporting. (Positive and negative)
	**Medium of PSE communication**				Medium of PSEs reporting. Use this broad category for broad statements
		Email			Staff report PSEs to leadership via email OR PSE response by email
		Hard Copy			Staff report PSEs to leadership by hard copy OR response by hard copy
		PSLS			Staff report PSEs via PSLS OR reponse by PSLS
		Verbal			Staff report PSEs verbally OR response verbally
		Other			Staff report PSEs other means not captured above OR response by other means
	**Events reported**				Types of events that are actually reported currently (not ones that SHOULD BE)

Name of Code	Node	Sub-node	Sub-node	Sub-node	Description
**Optimal state of PSE reporting**					General statements, not captured or not relevant to a single subcategory
	**Factors affecting reporting behaviour**				Ideal future systemic factors that encourage reporting. General.
		ED culture and leadership			Ideal future social behavior/norms/habits to encourage reporting.
		Clarity of Process			Ideal future process training/clarity to encourage reporting.
		User-friendliness of Process			Ideal future user experience to encourage reporting.
		Time Burden and Priorities			Ideal future time required and influence of priorities to encourage reporting.
		Value of reporting			Ideal future method to create value/utility across all staff to encourage reporting.
			Front Line		Ideal future method to create value/utility to encourage reporting @ front line.
				Feedback	Ideal future feedback to reporter to encourage reporting @ front line.
				Action	Ideal future actions resulting from reports to encourage reporting @front line.
				Data	Ideal future data sharing/utilzation to encourage reporting @ front line.
			Leadership		Ideal future value/utility of reporting to depart. leadership to encourage reporting
		Potential consequences			Ideal management of potential consquences to encourage reporting @front-line.
	**Medium of PSE communication**				Ideal future reporting medium and response medium.
	**Reportable Events**				Events that should be reported in the future state.
Gold Quotes					Quotes that captured key elements of the structure
Unprovoked Statements					Insightful, unprovoked statements
