# Supplementary material for: Beliefs and perceptions of patient safety event reporting in a Canadian Emergency Department: a qualitative study
Source: CJEM. 2022 Nov 7;24(8):867–75. doi: 10.1007/s43678-022-00400-2 (PMC9763130; doi:10.1007/s43678-022-00400-2)
Supplement: Supplementary file 3 — Supplementary file3 (PDF 81 kb) [file 43678_2022_400_MOESM3_ESM.pdf]

Appendix

Events deemed reportable by staff and associated examples.

Event	N	Example
Medication error	40	Wrong medication, wrong patient, wrong dose, pump programmed incorrectly
Interdepartmental operational challenges	32	Critical biochemical result not communicated from lab, incorrect radiology reporting, delayed imaging, patient transfer out of ED done incorrectly, infection control compliance, delayed specialist consultation, inappropriate specialist referral
Fall	26	Elderly falling out of bed or while mobilizing
Violence	19	Patient violence towards staff
ED operational challenges	13	Nurse has too many critically ill patients, staffing levels, out of catchment ambulance
Inappropriate care space	10	Inappropriate care space for infection control, inappropriate space for psychiatric patient, in appropriate care space for sick patient (waiting room)
Non-compliance with pre-established clinical pathway	9	Hot stroke pathway, sepsis pathway, STEMI pathway, blood transfusion process, delayed STAT orders, improper triage
Equipment issue or misuse	7	Pump programming, broken laryngoscope blade, needed equipment not available
ED communication error	6	Patient discharged when not ready for discharge, verbal order misunderstood, delayed orders completion
Wrong patient	1	Imaging, bloodwork on wrong patient
